# Effects of Cytokinins on Morphogenesis, Total (Poly)Phenolic Content and Antioxidant Capacity of In Vitro-Cultured Hop Plantlets, cvs. Cascade and Columbus

**DOI:** 10.3390/plants14030418

**Published:** 2025-01-31

**Authors:** Leandra Leto, Valeria Guarrasi, Anna Agosti, Martina Nironi, Benedetta Chiancone, Jorge Juan Vicedo

**Affiliations:** 1 Department of Food and Drug, University of Parma, Viale Parco Area delle Scienze 27/A, 43124 Parma, Italy; leandra.leto@unipr.it (L.L.); anna.agosti@unipr.it (A.A.); martina.nironi@unipr.it (M.N.); 2 Institute of Biophysics, National Research Council (CNR), Via Ugo La Malfa 153, 90146 Palermo, Italy; valeria.guarrasi@ibf.cnr.it; 3 Instituto de Investigación en Medio Ambiente y Ciencia Marina IMEDMAR, Universidad Católica de Valencia San Vicente Mártir, Carrer Guillem de Castro, 94, 46001 València, Spain; jorge.juan@ucv.es

**Keywords:** bioactive compounds, growth regulators, *Humulus lupulus* (L.), in vitro propagation, secondary metabolism

## Abstract

(1) Background: *Humulus lupulus* L. plants constitute a rich source of bioactive compounds. The synthesis of bioactive compounds in plants is often triggered by the activation of secondary metabolism, which can be induced by biotic or abiotic elicitors. In vitro, the effect of the elicitors can be studied in a controlled environment and in a small space, independently of seasonal variations. Cytokinins are frequently used in plant tissue culture for bud regeneration, branching and shoot elongation due to their role in cell division enhancement. This study aimed to investigate the effects of different cytokinins on the growth parameters, total (poly)phenolic content and antioxidant capacity of in vitro-grown hop plants to evaluate hop vitro-derived biomass as a potential source of bioactive compounds. (2) Methods: unimodal hop (cvs. Cascade and Columbus) explants were cultured on media enriched with four cytokinins (kinetin, 6-benzylaminopurine, meta-topolin and 6-(γ,γ-dimethylallylamino)-purine) at four concentrations. (3) Results: A genotype-dependent response to different cytokinins was encountered. (4) Conclusions Columbus explants could root in culture media auxin-free, providing valuable opportunities for commercial nurseries. Moreover, cytokinins were confirmed to be valuable elicitors to stimulate the bioactive compound biosynthesis in micropropagated hop plants, making them a precious source for various industries.

## 1. Introduction

Plant natural products (including phenolic compounds, flavonoids, terpenes, etc.) are attracting the attention of various industries, particularly in the agri-food, pharmaceutical and cosmetics sectors, which are increasingly interested in their application as plant-derived additives due to the wide range of remarkable bioactivities that they possess such as the antioxidant and antimicrobial ones, among others [1]. Hop (*Humulus lupulus* L.) plants are considered a highly valuable resource due to their rich composition in bioactive compounds that fulfill the criteria of bioactivities for the above-mentioned industries [2,3,4,5].

Hops are cultivated globally, including in Italy, primarily for their cones, which are a key ingredient in beer production [6]. In addition, extracts from seeds and cones present important antioxidant, cytotoxic and antimicrobial activity and, particularly those from cones, are also used in traditional medicine for treating sleeplessness, nervousness, anxiety, spasms, cough, fever, toothache and inflammation [7,8]. The bioactive properties of hops are primarily attributed to their content in secondary metabolites, such as (poly)phenols (e.g., catechin, epicatechin, quercetin, rutin, coumarin, gentisic acid, caffeic acid, ferulic acid, sinapic acid), α- and β-bitter acids (humulone, cohumulone, lupulone, colupulone) and terpenoids (e.g., β-myrcene, caryophyllene, humulene, β-farnesene, α- and β-selinene) [9,10]. These compounds are primarily produced in the lupulin glands and are concentrated in the glandular trichomes of hop cones, although they are also found in the leaves and stems of the plant [11,12].

As noted by Čeh [13], the types and quantities of these bioactive compounds can vary depending on environmental factors, such as seasonal changes, which can influence the plant’s secondary metabolism [14,15]. Additionally, the accumulation of (poly)phenols, bitter acids and xanthohumol in different plant parts may be influenced by hop variety [13]. One approach to increase the production of biomolecules in in vitro-cultured plants is to trigger their secondary metabolism by adding various elicitors to the growth media [16].

Recent studies have shown that in vitro cultures of hop plants can produce the same bioactive substances as plants grown in the field, such as (poly)phenols (flavan-3-ols, flavonols, prenylflavonoids, hydroxybenzoic acids and hydroxycinnamic acids), as well as α- and β-acids; in addition, the controlled conditions of in vitro culture techniques offer advantages such as genetic stability, efficient use of space and consistent, reliable production [17,18,19]. Murashige and Skoog (MS) basal medium [20] is widely used in plant tissue culture, typically supplemented with plant growth regulators (PGR) such as auxins, cytokinins, abscisic acid, gibberellins, ethylene and other substances with similar metabolic effects. Specifically, cytokinins play a key role in the regulation of the cell cycle and have an impact on plant growth and morphogenesis during in vitro regeneration [21,22,23]. Also, cytokinins contribute to maintaining the vitality of cultivated tissue by delaying senescence [24,25]. In addition, exogenous application of cytokinins in vitro can stimulate the secondary metabolism of hop plants, leading to the increased production of bioactive compounds [26].

In order to produce hop vitro-derived biomass to be used as a potential source of bioactive compounds, in this study, the effects of different cytokinins on the growth parameters and chemical composition of in vitro-grown hop plants, cvs. Cascade and Columbus, were investigated. These two cultivars were selected because of their importance on a global level. Cascade is an American aroma hop variety that was developed in 1972 and has since become one of the most widely grown hop varieties in the world. Cascade cones are known for their distinct floral and fruity aroma and are highly valued in the brewing industry as they give beer a unique character. Over the years, Cascade has become a staple in many craft beer recipes and continues to be a favorite among brewers worldwide [27,28]. Columbus, on the other hand, also known as Tomahawk or CTZ (Columbus/Tomahawk/Zues), is a high-alpha American hop variety that has gained great popularity since its introduction in the late 1990s. Columbus is known for its strong, pungent aroma and high bitterness and is commonly used in a variety of beer styles, especially those that emphasize hoppy bitterness and aromatic intensity [29].

## 2. Results

A statistical analysis revealed significant interactions between genotype and treatment (Table 1 and Table S1). Columbus explants showed comparable viability, independently of the cytokinin present in the culture medium (on average, 82.71% ± 29.75), while for cv. Cascade, the viability of explants was influenced by the type of cytokinin tested, with the explants cultured on 6-(γ,γ-Dimethylallylamino)purine (2iP) and meta-topolin (MT) (99.17 ± 3.63% and 95.00% ± 21.79, respectively) showing values statistically higher than those cultured on kinetin (KIN) (Figure S1).

Analyzing the data regarding the percentage of explants that produced sprouts and roots, a significant interaction among all factors was observed (Table 1 and Table S1). The two cultivars responded differently to the cytokinins in the culture medium for the parameter of the percentage of sprouting and rooting. In fact, for Cascade, the percentage of explants with sprouts was 55.42% ± 28.60 and the percentage with roots was 11.46% ± 19.82, regardless of the type and concentration of cytokinin used. In contrast, significant differences were observed in the percentage of sprouting only within explants grown on 6-Benzylaminopurine (BAP) enriched media for cv. Columbus. In fact, the highest significant percentage of sprouted explants was observed in BAP 2 μM, while the lowest was observed in the concentration of BAP 3 μM (Figure S2). When the percentage of rooted explants was considered, a trend could be observed: a decrease in the percentage of rooted explants is associated with the increase in kinetin concentration treatment, while for BAP and meta-topolin, rooting was completely inhibited with increasing cytokinin concentration. Only for 2iP, no statistical differences in the rooting response were recorded.

The formation of callus is regarded as a negative characteristic for the micropropagation of plants. In particular, in Cascade explants, the percentage of explants with callus decreased significantly when kinetin was present in the medium, compared to the other cytokinins considered; a different behavior was observed in Columbus, where callus production was independent of the type of cytokinin in the culture medium.

The number of sprouts produced by each explant was significantly different, depending on all the factors considered. In fact, for cv. Cascade, an average of 0.51 ± 0.1 sprouts per explant was recorded, independently of the cytokinin and its concentration. For Columbus, the highest significant number of sprouts was observed when explants were cultured on the media without cytokinin and the media enriched with cytokinin at 2 µM, independently of the type (Figure S3).

Finally, as expected, the presence of the cytokinins reduced the rhizogenesis for all the explants (Table 1 and  Table S1). As for other parameters tested, Cascade explants did not respond differently to the different cytokinins; instead for Columbus, a statistically higher number of roots (1.58 ± 1.5) was detected for the explants cultured on 2iP enriched media, in comparison to the other media tested.

Regarding the total (poly)phenolic content (TPC) values, the two cultivars responded differently: Columbus showed higher TPC values than Cascade, both considering the type of cytokinin and their concentration (Table 2 and  Table S2). Except for the 0 µM, the only cytokinin that, at other concentrations, did not influence the TPC parameter for both cultivars was kinetin (6.40 ± 0.76 mg GAE/g and 5.54 ± 0.39 mg GAE/g average, respectively) Within each cultivar, for Cascade, the TPC parameter was not influenced neither by the type nor the concentration of cytokinin, while for Columbus, the highest TPC values were registered in explants grown on media enriched with MT at the lowest concentration, or with 2iP at the highest. (Figure S4). These results were confirmed by the 2,2-diphenyl-1-picrylhydrazyl (DPPH) assay, in which Columbus had a higher content of molecules with antioxidant capacity compared to Cascade (5.17 ± 1.37 mg Trolox equivalent antioxidant capacity (TEAC)a/mL and 2.79 ± 1.19 mg TEAC/mL, respectively) (Figure S5). The 2,2′-azinobis(3-ethylbenzothiazoline-6-sulfonic acid (ABTS+) assay showed a significant statistical interaction between the factors “Genotype” × “Growth Regulator Concentration” and “Growth Regulator” × “Growth Regulator Concentration” (Table 2 and Table S2). Only for cv. Columbus, a significant influence of factors tested was observed; moreover, regardless of the genotype considered, a significant interaction between the factors “Growth regulator” and “Growth regulator concentration” was detected (Figure S6).

## 3. Discussion

The hop plant is widely recognized as a valuable source of bioactive compounds in in vitro culture systems, where it has been shown to grow optimally on a Murashige and Skoog medium (MS); moreover, this growth is further enhanced by the addition of elicitors, which are important not only for the growth and development of the cultured plant but also for the biosynthesis of secondary metabolites [17,19,30,31]. Elicitors are known to act as external signals that trigger various physiological and metabolic responses in plants. In this study, the influence of different cytokinins (and different concentrations) on growth parameters during the in vitro morphogenesis of hop cultures was studied. Also, the impact of the PGR type and concentration on the biosynthesis of (poly)phenols and on the antioxidant capacity of vitro-derived hop plantlets was determined.

The results obtained showed that all cytokinins were able to promote the proliferation of hop plantlets during the in vitro culture as practically all concentrations tested significantly affected the growth parameters measured (Table 1). Moreover, even if a quali-quantitative analysis of the bioactive compounds present in the hop vitro-derived plantlets was not performed, this study highlighted the influence of the type and concentration of cytokinins in the in vitro biosynthesis of secondary metabolites (Table 2). The same findings were reported for different species, such as *Scutellaria alpina* and *Stevia rebaudiana*, in which the bioactive compound profile of vitro-derived shoots was influenced by the type and concentration of the plant growth regulator used [32,33]. However, the extent of these effects depends on several factors, among which the genotype studied normally plays a crucial role [17,19]. In terms of genotype-specific responses, in this study, it is reported that the viability of the Columbus plantlets was largely unaffected by the cytokinin presence in the culture medium, whereas the viability of the Cascade genotype decreased significantly in the presence of kinetin, particularly in its higher concentrations. These results are consistent with the previous findings of Clapa [34] and Mafakheri [35], who reported that, in hops, a genotype-dependent response to cytokinins was individuated.

In addition, the results showed that the highest percentage of explants that formed both shoots and roots was obtained with lower concentrations of BAP, especially in the cultivar Columbus. This result is consistent with the studies of Batista [36] on hops, who found that lower cytokinin concentrations were more effective in promoting shoot and root development in other plant species. This observation underscores the importance of individuating the right cytokinin and optimizing cytokinin concentrations to avoid inhibitory effects on plant growth that can occur at higher concentrations. Moreover, the 2iP determined the formation of a higher number of roots, when compared to the other cytokinins tested. This result is particularly interesting since, as reported by other authors [30,34], the addition of auxins in the culture medium is mandatory for inducing rhizogenesis. Cytokinins are widely recognized for their ability to promote cell division, tissue differentiation and high rates of morphogenesis at the right doses [30,37,38,39,40]. In particular, higher concentrations of cytokinins not only inhibit shoot development but also impede root formation during the plant in vitro regeneration, as reported in several species, among which are *Arabidopsis thaliana* and *Oryza sativa* [41,42,43,44]. On the contrary, in this study, a relatively high rooting response is obtained, without the use of auxins and with different cytokinins in the culture medium. This achievement will allow for a reduction in the time and costs of hop micropropagation, aspects particularly interesting for commercial nurseries. The different responses obtained in this study compared with those reported by other authors may be due to different plant hormonal signaling networks, which can modify the normal growth processes [45]. In addition, high cytokinin concentrations have been associated with the induction of callus formation, which, although useful in some contexts, is generally considered an undesirable outcome in micropropagation systems as it can divert resources away from desired organogenesis [23]. In this study, in agreement with the cited studies, callus formation did not occur in explants grown in the cytokinin-free medium; it increased significantly with the increase in cytokinin concentration in the medium, thus supporting the idea that high cytokinin concentrations can lead to undesired development of callus tissue.

The role of cytokinins as plant biostimulants goes beyond mere growth regulation: they also trigger various physiological and metabolic responses in plants [46], which are often induced by increased concentrations of elicitors or growth regulators. This increase in antioxidant capacity suggests that the plants are actively responding to oxidative stress or other environmental challenges created by the cytokinin treatments [47]. The higher values obtained for total (poly)phenolic content and antioxidant capacity in the extracts obtained from plantlets grown in a medium supplemented with low concentrations of some cytokinins or with high concentrations of others are consistent with the work of Singh on *Centella asiatica* [48] and of Damanik on *Glycine max* [47], which showed that the plant’s response to growth regulators is species-specific and highly dependent on the concentration of cytokinin used. This increase in (poly)phenol content and antioxidant capacity in Columbus confirms another assumption that specific cytokinin treatments at certain concentrations may be a promising strategy to increase the production of bioactive compounds in hop plants. This underscores the importance of optimizing cytokinin treatments to achieve the desired results in both growth promotion and secondary metabolite production.

## 4. Materials and Methods

### 4.1. Plant Material and Culture Conditions

For this study, in vitro-cultured plantlets of hops, cvs. Cascade and Columbus, derived from the collection of the University of Parma (Italy), were used. To establish the in vitro cultures, young sprouts, derived from hop rhizome, were sterilized and cultured in Microbox ECO_2_ containers (Combiness, Belgium—Micropoli, Italy) containing a solidified proliferation culture medium (PM) with the following ingredients: Murashige and Skoog (MS) basal salt mixture (1×), MS vitamin mixture (1×) as described by Murashige and Skoog [20], 30 g/L sucrose, as a carbon source and 8.0 g/L agar, as a gelling agent. The pH of the media was adjusted to 5.8 using NaOH or HCl (1 N) before autoclaving. Microbox containers containing the culture media were autoclaved at 121 °C for 20 min and 1 atmosphere of pressure.

### 4.2. Experimental Design

**Figure 1:**
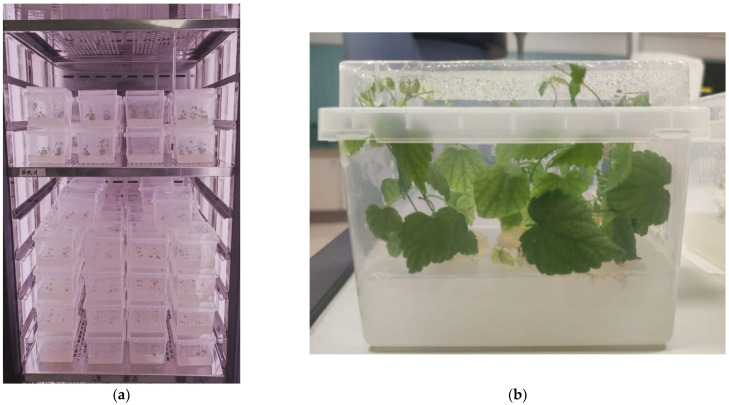
(**a**) Microbox ECO_2_ containers with hop, cv. Columbus, explants cultivated in the growth chamber, with different cytokinins; (**b**) hop plantlets cultured in vitro for four weeks.

The influence of cytokinins on morphogenesis and on secondary metabolite biosynthesis was studied on explants (uninodal microcuttings) taken from in vitro cultures of Cascade and Columbus cultivars. Four concentrations (1, 2, 3 and 4 µM) of the following cytokinins were tested KIN, BAP, MT and 2iP. Replicates containing explants cultured without cytokinins served as controls. These concentrations were selected within the typical range of cytokinin concentrations generally recommended for plant tissue culture. Six uninodal microcuttings were cultured per each Microbox, and an overall amount of 4 Microboxes per treatment were disposed of in the growth chamber (EGCS 701 3S-Equitec, Madrid, Spain) (Figure 1a). The culture conditions included a constant temperature of 25 ± 1 °C, with photosynthetically active radiation of 20 μmol m^−2^s^−1^, in a 16 h photoperiod, provided by linear fluorescent lamps with white daylight illumination (8500 K, T8 Gro-lux fluorescent tubes, Sylvania Lamps, Erlangen, Germany) for four weeks (Figure 1b).

To assess the impact of the above-mentioned cytokinins on morphogenesis, the following parameters were measured at the end of the experiment: viability (%) (number of viable explants after 4 weeks × 100)/total number of explants; sprouting (%) (number of sprouted explants after 4 weeks × 100)/total number of explants; rooting (%) (number of rooted explants after 4 weeks × 100)/total number of explants; callus (%) (number of explants with callus, formed after 4 weeks × 100)/total number of explants; and number (n°) of sprouts and roots per explant.

### 4.3. Biochemical Analysis

#### 4.3.1. Sample Extraction

To perform the biochemical analysis, sample extraction was performed following the protocol reported by Leto et al. [19]. In detail, the biomass obtained was freeze-dried using a Scanvac Coolsafe 4 l freeze dryer (LaboGene, Lillerød, Denmark). The dried plantlets were then pulverized and subjected to extraction with ethanol/water (EW) solution (80/20 *v*/*v*). A dilution factor of 1:20 was used during the extraction process. The extraction was carried out in a shaker (orbital shaker, LBX Orb-B2, Premia de Dalt, Barcelona, Spain) at 200 strokes per minute for 2 h at the lab temperature (≈21 °C). After extraction, samples were centrifuged for 10 min at 5000 rpm at the lab temperature (Thermo Scientific Sorvall ST1 Plus, Thermo Electron LED GmbH, Osterode am Harz, Germany) to separate the supernatants. These supernatants were then further diluted in a 1:5 ratio with distilled water. Each extraction procedure was repeated in triplicate to determine total (poly)phenolic content using the Folin–Ciocalteu assay, and antioxidant capacity (AO) using DPPH and ABTS+ assays.

#### 4.3.2. Determination of Total (Poly)Phenolic Content

TPC of the plant extracts was determined using a modified version of the protocol described by Onder [49].

For the determination of TPC, 250 μL of the plant extract was mixed with 1 mL of Folin–Ciocalteu phenol reagent that had been previously diluted in double-distilled water (1:10 *v*/*v*). The Folin–Ciocalteu reagent is often used for the detection of phenolic compounds as it reacts with the hydroxyl groups of the (poly)phenols and forms a blue-colored complex that can be quantified spectrophotometrically. To neutralize the reaction and to promote the formation of the blue complex, 2 mL of a 10% (*w*/*v*) sodium carbonate solution was added. The mixture was then incubated for 30 min in the dark at the lab temperature (≈21 °C) to allow the reaction of phenolic compounds with the reagent in the extract.

The absorbance of the resulting solution was measured at 760 nm using a JASCO V-530 spectrophotometer (Easton, MD, USA). This wavelength corresponds to the absorption maximum of the blue complex formed between the phenolic compounds and the Folin–Ciocalteu reagent.

To calculate the TPC, a calibration curve was created using gallic acid as a standard (Sigma-Aldrich, St. Louis, MO, USA). Gallic acid, a commonly used reference compound due to its high phenolic content, was prepared in a concentration range of 10–100 mg/L (5 points), and the absorbance of each concentration was measured under the same experimental conditions. The TPC of the plant extracts was then determined by comparing the absorbance values of the samples with the calibration curve, and the results were expressed as milligrams of gallic acid equivalent per gram of dry matter (mg GAE/g DM). This value represents the (poly)phenolic content relative to the standard gallic acid, which serves as a guide to the antioxidant capacity of the samples.

This test provides a reliable and efficient means of quantifying total (poly)phenolic content, which is a key indicator of the antioxidant activity and potential health benefits of plant material.

#### 4.3.3. Antioxidant Capacity of Hop Vitro-Derived Vegetative Biomass, Through DPPH and ABTS+ Assays

To evaluate the radical scavenging activity of the different hop biomass, the DPPH assay was performed according to the method described by Abram [14] with slight modifications. The DPPH assay is a widely used technique to measure the ability of compounds to neutralize free radicals, which is an important indicator of their antioxidant potential. In this method, 2.9 mL of an ethanolic DPPH solution (0.05 mM) was prepared and 100 μL of the plant extract sample was added to this solution. The mixture was then incubated for 30 min at the lab temperature in the dark to allow the reaction to proceed. The DPPH radical, which has a deep purple color, is reduced to a light-yellow hue when it interacts with an antioxidant, and this color change corresponds to the scavenging activity of the sample.

After the incubation period, the absorbance of the reaction mixture was measured at 517 nm using a JASCO V-530 spectrophotometer, with three replicates for each sample to ensure accuracy. A blank control, consisting of 100 μL of the extraction solution without the sample, was prepared and measured under the same conditions to account for any background absorbance.

Trolox (Sigma-Aldrich, St. Louis, MO, USA), a water-soluble vitamin E analog, was used as a reference standard to generate a calibration curve. The Trolox concentration ranged from 0.1 mM to 1 mM (5 points) and allowed quantification of the antioxidant capacity of the extracts. The radical scavenging capacity of the extracts was expressed as the percentage of inhibition (I%) of the DPPH radical. This percentage was calculated based on the difference in absorbance between the control (without extract) and the sample (with extract). The results were then converted to mM TEAC (Trolox equivalent antioxidant capacity), which is a standardized measure of antioxidant capacity compared to Trolox.

The ABTS+ assay was performed to evaluate the antioxidant capacity of the plant extracts, following the method described by Wu [50], with slight modifications. The ABTS+ assay is a widely used method for evaluating the free radical scavenging ability of compounds and is particularly suitable for determining the antioxidant capacity of both hydrophilic and lipophilic substances.

To prepare the ABTS+ radical solution, 7 mM ABTS+ solution was mixed with 2.45 mM potassium persulfate, and the resulting mixture was kept in the dark for about 16 h to allow the formation of the ABTS+ radical. This radical is very stable and has a characteristic green-blue color that can be monitored spectrophotometrically. After the incubation period, the ABTS+ solution was diluted with ethanol at a ratio of 1:70 (*v*/*v*) and the concentration of the radical was adjusted to achieve an absorbance of 0.7 ± 0.2 at 734 nm, which is the optimal wavelength for detection.

For the assay, 100 μL of the plant extract sample (or a blank control or Trolox standard solution) was mixed with 1900 μL of the ABTS+ working solution. The reaction was allowed to run at the lab temperature in the dark for a period of time to avoid any light-induced interference, as the absorbance of the radical can be affected by exposure to light. After the reaction, the absorbance of each sample was measured at 734 nm using a JASCO V-530 spectrophotometer (Easton, MD, USA).

The antioxidant capacity of the samples was quantified by comparing the reduction in absorbance caused by the antioxidant capacity of the plant extracts with that of Trolox, a water-soluble vitamin E analog, which serves as a reference standard. The degree of absorption inhibition was calculated as a percentage, allowing an estimation of the radical scavenging capacity of the extracts. Trolox concentrations were used to generate a calibration curve, as described in the DPPH assay, and the results were expressed as TEAC.

For both assays, the concentration of the sample was converted from millimolar (mM) to mg/mL by applying the dilution factor and the molecular weight of the compound.

This method provides a reliable approach to evaluating the overall antioxidant potential of plant extracts by measuring their ability to neutralize the ABTS+ radical, a stable, water-soluble radical that mimics the capacity of more complex radicals in biological systems.

### 4.4. Statistical Analyses of Data

Data were expressed as means ± standard deviations (SD). The data obtained from the growth parameters and the biochemical analysis, after having evaluated their normal distribution by the Kolmogorov–Smirnov’s test, were statistically evaluated using a three-way analysis of variance (ANOVA). The ANOVA considered three factors: “Genotype (G)”, “Growth Regulator (GR)” and “Growth Regulator Concentration (GRC)”. Tukey’s honest significance test (HSD) was used to separate the means at the 5% level of significance (*p* ≤ 0.05) using the Systat Software (SYSTAT 13.1, Systat Software, Inc.; Pint Richmond, CA, USA).

## 5. Conclusions

The present study demonstrates that the plant cytokinins kinetin, BAP, meta-topolin and 2iP have a significant impact on hop morphogenesis and (poly)phenol biosynthesis in vitro.

A strong influence of genotype was observed in the response of explants to various types and concentrations of cytokinins. While Cascade did not show statistically significant differences between the control and the medium of different cytokinins and different concentrations, with effects limited to viability, Columbus, although with differences, showed positive effects on all growth parameters considered. Remarkably, a satisfactory rhizogenic response was achieved in Columbus even without the use of auxins during the micropropagation process. This finding opens new possibilities for commercial nurseries, especially for those who want to reduce the use of growth regulators and minimize the time required for large-scale propagation of hop plants.

The same genotype-dependent response was observed when the total (poly)phenolic content produced by both cultivars was analyzed. In fact, only in the plantlets of Columbus did the production of biomolecules increase depending on the type and cytokinins used.

In light of the findings of this study, further research is warranted to explore the potential of vitro-derived hop biomass as a source of bioactive compounds. In future research, hop plantlets at different developmental stages will be analyzed; furthermore, a more comprehensive analysis will be conducted at the molecular level to ascertain the impact of the type and concentration of plant growth regulators on the evolution of bioactive compounds in in vitro-cultured hop explants.

## Figures and Tables

**Table 1:** Influence of genotype, type and concentration of cytokinins on hop plantlet growth parameters, after four weeks of culture.

Genotype (G)	Growth Regulator (GR)	Growth Regulator Concentration (GRC)	Viability		Sprouting		Rooting		Callus		Sprouts		Roots	
			%	±SD	%	±SD	%	±SD	%	±SD	n°	±SD	n°	±SD
CASCADE	Kinetin	0 µM	100.00	0.00	45.83	13.82	41.67	14.43	0.00	0.00	0.67	0.12	0.88	0.25
		1 µM	58.33	8.33	41.67	8.33	0.00	0.00	33.33	20.41	0.67	0.20	0.00	0.00
		2 µM	62.50	13.82	41.67	30.05	0.00	0.00	41.67	30.05	0.75	0.67	0.00	0.00
		3 µM	62.50	7.22	29.17	24.65	0.00	0.00	50.00	11.79	0.42	0.38	0.00	0.00
		4 µM	66.67	11.79	33.33	23.57	0.00	0.00	45.83	13.82	0.46	0.36	0.00	0.00
	BAP	0 µM	100.00	0.00	45.83	13.82	41.67	14.43	0.00	0.00	0.67	0.12	0.88	0.25
		1 µM	100.00	0.00	66.67	16.67	8.33	14.43	100.00	0.00	0.75	0.28	0.13	0.22
		2 µM	70.83	29.76	50.00	11.79	0.00	0.00	75.00	25.00	0.67	0.20	0.00	0.00
		3 µM	87.50	13.82	54.17	13.82	0.00	0.00	75.00	27.64	0.00	0.00	0.00	0.00
		4 µM	79.17	13.82	58.33	18.63	0.00	0.00	83.33	11.79	0.25	0.28	0.00	0.00
	Meta-topolin	0 µM	100.00	0.00	45.83	13.82	41.67	14.43	0.00	0.00	0.67	0.12	0.88	0.25
		1 µM	75.00	43.30	75.00	43.30	8.33	14.43	75.00	43.30	0.46	0.49	0.00	0.00
		2 µM	100.00	0.00	100.00	0.00	0.00	0.00	100.00	0.00	0.29	0.25	0.00	0.00
		3 µM	100.00	0.00	100.00	0.00	0.00	0.00	100.00	0.00	0.08	0.14	0.00	0.00
		4 µM	100.00	0.00	100.00	0.00	0.00	0.00	100.00	0.00	0.46	0.79	0.00	0.00
	2iP	0 µM	100.00	0.00	45.83	13.82	41.67	14.43	0.00	0.00	0.67	0.12	0.88	0.25
		1 µM	100.00	0.00	33.33	11.79	12.50	21.65	100.00	0.00	0.42	0.14	0.17	0.29
		2 µM	95.83	7.22	58.33	14.43	25.00	25.00	95.83	7.22	0.63	0.14	0.38	0.38
		3 µM	100.00	0.00	54.17	18.16	8.33	14.43	100.00	0.00	0.88	0.32	0.08	0.14
		4 µM	100.00	0.00	29.17	18.16	0.00	0.00	91.67	14.43	0.33	0.20	0.00	0.00
COLUMBUS	Kinetin	0 µM	87.50	13.82	79.17	13.82	58.33	8.33	0.00	0.00	1.25	0.28	2.00	0.49
		1 µM	87.50	21.65	87.50	21.65	8.33	14.43	66.67	20.41	0.46	0.27	0.08	0.14
		2 µM	100.00	0.00	100.00	0.00	8.33	8.33	87.50	21.65	0.63	0.30	0.17	0.17
		3 µM	100.00	0.00	100.00	0.00	4.17	7.22	75.00	18.63	0.50	0.12	0.13	0.22
		4 µM	87.50	21.65	87.50	21.65	0.00	0.00	83.33	20.41	0.46	0.18	0.00	0.00
	BAP	0 µM	87.50	13.82	79.17	13.82	58.33	8.33	0.00	0.00	1.25	0.28	2.00	0.49
		1 µM	91.67	14.43	66.67	20.41	66.67	20.41	79.17	21.65	0.71	0.41	0.08	0.14
		2 µM	100.00	0.00	100.00	0.00	0.00	0.00	100.00	0.00	1.79	0.14	0.00	0.00
		3 µM	66.67	39.09	16.67	11.79	0.00	0.00	58.33	34.36	0.29	0.25	0.00	0.00
		4 µM	62.50	21.65	41.67	8.33	0.00	0.00	87.50	13.82	0.67	0.17	0.00	0.00
	Meta-topolin	0 µM	87.50	13.82	79.17	13.82	58.33	8.33	0.00	0.00	1.25	0.28	2.00	0.48
		1 µM	70.83	29.76	62.50	24.65	79.17	21.65	100.00	0.00	0.63	0.32	0.00	0.00
		2 µM	62.50	24.65	62.50	24.65	0.00	0.00	95.83	7.22	1.17	0.42	0.00	0.00
		3 µM	100.00	0.00	100.00	0.00	0.00	0.00	100.00	0.00	0.58	0.08	0.00	0.00
		4 µM	75.00	43.30	75.00	43.30	0.00	0.00	75.00	43.30	0.58	0.42	0.00	0.00
	2iP	0 µM	87.50	13.82	79.17	13.82	58.33	8.33	0.00	0.00	1.25	0.28	2.00	0.48
		1 µM	75.00	43.30	75.00	43.30	29.17	29.76	45.83	46.21	0.54	0.62	1.58	1.65
		2 µM	75.00	43.30	75.00	43.30	41.67	43.30	66.67	40.82	0.88	0.66	1.54	2.13
		3 µM	75.00	43.30	75.00	43.30	41.67	43.30	75.00	43.30	0.79	0.59	1.79	1.86
		4 µM	75.00	43.30	66.67	40.82	41.67	25.00	66.67	40.82	0.83	0.60	0.96	0.56
Statistical analysis of data			*p*		*p*		*p*		*p*		*p*		*p*	
G			0.186		<0.001		<0.001		0.957		<0.001		<0.001	
GR			0.593		<0.001		<0.001		<0.001		0.558		<0.001	
GRC			0.242		0.340		<0.001		<0.001		<0.001		<0.001	
G × GRC			0.709		0.486		0.002		0.793		0.030		0.054	
GR × G			0.001		<0.001		0.006		<0.001		0.096		0.001	
GRC × GR			0.625		0.049		<0.001		0.319		0.052		0.690	
G × GR × GRC			0.437		0.021		<0.001		0.211		0.546		0.921	

Three-way analysis of variance (ANOVA), Tukey’s test (*p* ≤ 0.05). Abbreviations: BAP: 6-Benzylaminopurine; 2iP: 6-(γ,γ-Dimethylallylamino)purine.

**Table 2:** Influence of genotype, type and concentration of cytokinins on hop plantlet biochemical parameters, after four weeks of culture.

Genotype (G)	Growth Regulator (GR)	Growth Regulator Concentration (GRC)	TPC		DPPH		ABTS+	
			mg GAE/g	±SD	mg TEAC/mL	±SD	mg TEAC/mL	±SD
CASCADE	Kinetin	0 µM	5.36	0.02	2.14	0.21	16.25	0.83
		1 µM	6.09	0.15	2.04	0.64	20.29	2.71
		2 µM	6.04	0.15	2.85	0.26	22.54	0.46
		3 µM	5.98	0.16	3.83	0.00	16.50	1.50
		4 µM	5.80	0.14	2.65	0.62	18.84	3.00
	BAP	0 µM	5.36	0.02	2.14	0.21	16.25	0.83
		1 µM	5.35	0.01	3.06	0.11	26.09	7.00
		2 µM	5.00	0.17	1.76	0.46	15.21	3.04
		3 µM	5.34	0.04	1.62	0.05	19.04	1.79
		4 µM	5.31	0.15	2.50	0.11	19.09	2.58
	Meta-topolin	0 µM	5.36	0.02	2.14	0.21	16.25	0.83
		1 µM	5.67	0.06	2.16	0.03	20.71	1.29
		2 µM	5.84	0.32	1.22	0.18	19.88	1.29
		3 µM	5.35	0.09	2.11	0.15	17.67	0.75
		4 µM	5.98	0.10	2.57	0.21	15.25	3.75
	2iP	0 µM	5.36	0.02	2.14	0.21	16.25	0.83
		1 µM	5.03	0.01	4.09	0.20	9.25	3.17
		2 µM	5.99	0.23	4.91	0.92	12.75	0.33
		3 µM	5.74	0.09	5.81	0.11	14.29	1.38
		4 µM	4.86	0.16	4.03	0.33	13.21	0.63
COLUMBUS	Kinetin	0 µM	6.87	0.02	4.98	0.26	18.25	0.33
		1 µM	5.65	0.21	3.57	0.03	24.84	0.33
		2 µM	5.70	0.07	4.17	0.15	16.63	4.63
		3 µM	6.35	0.47	4.44	0.31	19.88	2.13
		4 µM	5.18	0.04	3.44	0.07	11.12	2.04
	BAP	0 µM	6.87	0.02	4.98	0.26	18.25	0.33
		1 µM	6.91	0.03	6.58	0.16	20.79	0.71
		2 µM	6.23	0.01	5.43	0.16	16.04	3.71
		3 µM	5.67	0.03	4.76	0.18	14.29	2.71
		4 µM	4.83	0.24	3.14	0.49	12.58	0.75
	Meta-topolin	0 µM	6.87	0.02	4.98	0.26	18.25	0.33
		1 µM	7.49	0.15	7.50	0.07	14.38	4.46
		2 µM	6.99	0.37	5.25	0.51	15.63	2.29
		3 µM	5.93	0.02	4.27	0.02	12.88	0.46
		4 µM	5.68	0.07	4.24	0.21	10.37	1.13
	2iP	0 µM	6.87	0.02	4.98	0.26	18.25	0.33
		1 µM	6.54	0.05	5.91	0.38	10.79	0.63
		2 µM	6.59	0.07	5.32	1.36	10.58	0.25
		3 µM	7.22	0.05	7.89	0.26	11.21	1.79
		4 µM	7.54	0.03	7.55	0.02	9.37	2.29
Statistical analysis of data			*p*		*p*		*p*	
G			<0.001		<0.001		0.007	
GR			<0.001		<0.001		<0.001	
GRC			<0.001		<0.001		0.003	
G × GRC			<0.001		0.003		0.030	
GR × G			<0.001		<0.001		0.449	
GRC × GR			<0.001		<0.001		0.022	
G × GR × GRC			<0.001		<0.001		0.405	

Three-way analysis of variance (ANOVA), Tukey’s test (*p* ≤ 0.05). Abbreviations: BAP: 6-Benzylaminopurine; 2iP: 6-(γ,γ-Dimethylallylamino)purine. TPC: total (poly)phenolic content; DPPH: 2,2-diphenyl-1-picrylhydrazyl assay; ABTS+: 2,2′-azinobis(3-ethylbenzothiazoline-6-sulfonic acid assay.

## Data Availability

Data are contained within the article or Supplementary Materials.

## Supplementary Materials

The following supporting information can be downloaded at: https://www.mdpi.com/article/10.3390/plants14030418/s1, Table S1: Three-way ANOVA test to evaluate the significance of *Humulus lupulus* (L.) genotype, type of growth regulator and growth regulator concentration on plantlet growth parameters, after four weeks of culture; Table S2: Three-way ANOVA test to evaluate the significance of *Humulus lupulus* (L.) genotype, type of growth regulator and growth regulator concentration on plantlet biochemical parameters, after four weeks of culture; Figure S1: Effect of ‘type of cytokinin’ on the viability of cv. Cascade explants after four weeks of culture. Letters indicate statistically significant values according to the Tukey test post-two-way-ANOVA (‘Genotype’ × ‘Growth Regulator’) at *p* ≤ 0.05 (Table 1). Abbreviations: BAP: 6-Benzylaminopurine; 2iP: 6-(γ,γ-Dimethylallylamino)purine; Figure S2: Effect of BAP concentration on the percentage of sprouted explants of cv. Columbus explants after four weeks of culture. Letters indicate statistically significant values according to the Tukey test post-three-way-ANOVA (‘Genotype’ × ‘Growth Regulator’× ’Growth Regulator Concentration’) at *p* ≤ 0.05 (Table 1). Abbreviations: BAP: 6-Benzylaminopurine; Figure S3: Effect of the concentration of the growth regulators tested on the number of sprouts produced by each explant of cv. Columbus after four weeks of culture. Letters indicate statistically significant values according to the Tukey test post-two-way-ANOVA (‘Genotype’ × ’Growth Regulator Concentration’) at *p* ≤ 0.05 (Table 1); Figure S4: Effect of the type and concentration of the growth regulators tested on the total (poly)phenolic content of cv. Columbus plantlets after four weeks of culture. Letters indicate statistically significant values according to the Tukey test post-three-way-ANOVA (‘genotype’ × ‘Growth Regulator’× ’Growth Regulator Concentration’) at *p* ≤ 0.05 (Table 2). Abbreviations: BAP: 6-Benzylaminopurine, 2iP: 6-(γ,γ-Dimethylallylamino)purine; Figure S5: Effect of the type and concentration of the growth regulators tested on antioxidant capacity (measured with DPPH assay) of cvs. Cascade and Columbus plantlets after four weeks of culture. Between the two genotypes tested, letters indicate statistically significant values, according to the Tukey test post-three-way-ANOVA (‘Genotype’ × ‘Growth Regulator’× ’Growth Regulator Concentration’) at *p* ≤ 0.05 (Table 2). Abbreviations: BAP: 6-Benzylaminopurine, 2iP: 6-(γ,γ-Dimethylallylamino)purine, DW: dry weight; Figure S6: Effect of the type and concentration of the growth regulators tested on antioxidant capacity of cvs. Cascade and Columbus plantlets after four weeks of culture. Between the two genotypes tested, letters indicate statistically significant values, according to the Tukey test post-two-way-ANOVA (‘Growth Regulator’× ’Growth Regulator Concentration’) at *p* ≤ 0.05 (Table 2). Abbreviations: BAP: 6-Benzylaminopurine, 2iP: 6-(γ,γ-Dimethylallylamino)purine, DW: dry weight.
