# Supplementary material for: Effects of Cytokinins on Morphogenesis, Total (Poly)Phenolic Content and Antioxidant Capacity of In Vitro-Cultured Hop Plantlets, cvs. Cascade and Columbus
Source: Plants (Basel). 2025 Jan 31;14(3):418. doi: 10.3390/plants14030418 (PMC11820385; doi:10.3390/plants14030418)
Supplement: Supplementary file 1 [file plants-14-00418-s001.zip › plants-3410236-supplementary.pdf]

## Supplementary material

### Tables

**Table S1.** Three-way ANOVA test to evaluate the significance of *Humulus lupulus* (L.) genotype, type of growth regulator and growth regulator concentration on plantlet growth parameters, after four weeks of culture.

| Tested Parameters | Variation Source | Sum of Squares | F Value | *p* Value [Pr (>F)] |
|---|---|---|---|---|
| Viability | Genotype (G) | 1.085,069 | 1,767 | 0,186 |
| | Growth Regulator (GR) | 390,625 | 0,636 | 0,593 |
| | Growth Regulator Concentration (GRC) | 852,431 | 1,388 | 0,242 |
| | G × GRC | 329,861 | 0,537 | 0,709 |
| | GR × G | 3.839,699 | 6,254 | 0,001 |
| | GRC × GR | 506,366 | 0,825 | 0,625 |
| | G × GR × GRC | 625,000 | 1,018 | 0,437 |
| Sprouting | Genotype (G) | 16.000,000 | 24,686 | <0,001 |
| | Growth Regulator (GR) | 4.115,741 | 6,350 | <0,001 |
| | Growth Regulator Concentration (GRC) | 740,451 | 1,142 | 0,340 |
| | G × GRC | 561,632 | 0,867 | 0,486 |
| | GR × G | 7.199,074 | 11,107 | <0,001 |
| | GRC × GR | 1.191,840 | 1,839 | 0,049 |
| | G × GR × GRC | 1.367,188 | 2,109 | 0,021 |
| Rooting | Genotype (G) | 18.062,500 | 58,231 | <0,001 |
| | Growth Regulator (GR) | 2.708,333 | 8,731 | <0,001 |
| | Growth Regulator Concentration (GRC) | 10.549,479 | 34,010 | <0,001 |
| | G × GRC | 1.356,771 | 4,374 | 0,002 |
| | GR × G | 1.354,167 | 4,366 | 0,006 |
| | GRC × GR | 1.613,137 | 5,201 | <0,001 |
| | G × GR × GRC | 1.552,373 | 5,005 | <0,001 |
| Callus | Genotype (G) | 1,736 | 0,003 | 0,957 |
| | Growth Regulator (GR) | 4.779,514 | 7,934 | <0,001 |
| | Growth Regulator Concentration (GRC) | 40.223,958 | 66,769 | <0,001 |
| | G × GRC | 253,472 | 0,421 | 0,793 |

| | | | | |
|---|---|---|---|---|
| | GR × G | 5.057,292 | 8,395 | <0,001 |
| | GRC × GR | 699,653 | 1,161 | 0,319 |
| | G × GR × GRC | 800,926 | 1,329 | 0,211 |
| Sprouts | Genotype (G) | 4,011 | 24,337 | <0,001 |
| | Growth Regulator (GR) | 0,114 | 0,694 | 0,558 |
| | Growth Regulator Concentration (GRC) | 1,619 | 9,823 | <0,001 |
| | G × GRC | 0,456 | 2,769 | 0,030 |
| | GR × G | 0,357 | 2,166 | 0,096 |
| | GRC × GR | 0,300 | 1,819 | 0,052 |
| | G × GR × GRC | 0,149 | 0,903 | 0,546 |
| Roots | Genotype (G) | 10,167 | 24,034 | <0,001 |
| | Growth Regulator (GR) | 3,985 | 9,421 | <0,001 |
| | Growth Regulator Concentration (GRC) | 9,576 | 22,637 | <0,001 |
| | G × GRC | 1,012 | 2,393 | 0,054 |
| | GR × G | 2,655 | 6,276 | <0,001 |
| | GRC × GR | 0,321 | 0,760 | 0,690 |
| | G × GR × GRC | 0,205 | 0,484 | 0,921 |

Three-way analysis of variance (ANOVA), Tukey's test ($p \leq 0.05$). When the triple interaction 'Genotype × Growth Regulator × Growth Regulator Concentration' or the double interactions 'Genotype × Growth Regulator', 'Genotype × Growth Regulator Concentration' and 'Growth Regulator Concentration × Growth Regulator' were not significant for either of the traits, respectively, two-way  and one way- ANOVAs were conducted (Figure S1, S2 and S3).

**Table S2.** Three-way ANOVA test to evaluate the significance of *Humulus lupulus* (L.) genotype, type of growth regulator and growth regulator concentration on plantlet biochemical parameters, after four weeks of culture

| Tested Parameters | Variation Source | Sum of Squares | F Value | p Value [Pr (>F)] |
|---|---|---|---|---|
| TPC | Genotype (G) | 14,710 | 339,744 | < 0,001 |
| | Growth Regulator (GR) | 0,989 | 22,846 | < 0,001 |
| | Growth Regulator Concentration (GRC) | 0,579 | 13,376 | < 0,001 |
| | G × GRC | 0,840 | 19,397 | < 0,001 |
| | GR × G | 1,807 | 41,736 | < 0,001 |
| | GRC × GR | 0,459 | 10,600 | < 0,001 |
| | G × GR × GRC | 0,648 | 14,968 | < 0,001 |
| DPPH | Genotype (G) | 113,260 | 413,252 | < 0,001 |
| | Growth Regulator (GR) | 14,862 | 54,226 | < 0,001 |
| | Growth Regulator Concentration (GRC) | 2,059 | 7,514 | < 0,001 |
| | G × GRC | 1,329 | 4,849 | 0,003 |
| | GR × G | 3,038 | 11,084 | < 0,001 |
| | GRC × GR | 2,964 | 10,815 | < 0,001 |
| | G × GR × GRC | 1,512 | 5,517 | < 0,001 |
| ABTS+ | Genotype (G) | 84,961 | 8,149 | 0,007 |
| | Growth Regulator (GR) | 138,589 | 13,292 | < 0,001 |
| | Growth Regulator Concentration (GRC) | 48,943 | 4,694 | 0,003 |
| | G × GRC | 31,149 | 2,988 | 0,030 |
| | GR × G | 9,400 | 0,902 | 0,449 |
| | GRC × GR | 24,441 | 2,344 | 0,022 |
| | G × GR × GRC | 11,217 | 1,076 | 0,405 |

Three-way analysis of variance (ANOVA), Tukey's test ($p \leq 0.05$). When the triple interaction 'Genotype × Growth Regulator × Growth Regulator Concentration' or the double interactions 'Genotype × Growth Regulator', 'Genotype × Growth Regulator Concentration' and 'Growth Regulator Concentration × Growth Regulator' were not significant for either of the traits, respectively, two-way and one way-ANOVAs were conducted (Figure S4, S5 and S6).

## Figures

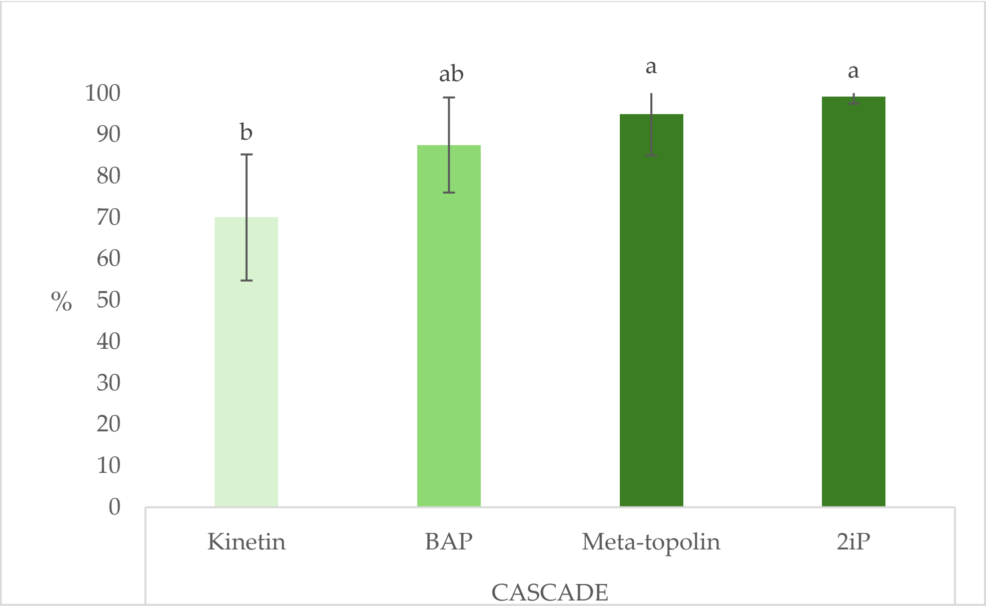


**Figure S1**

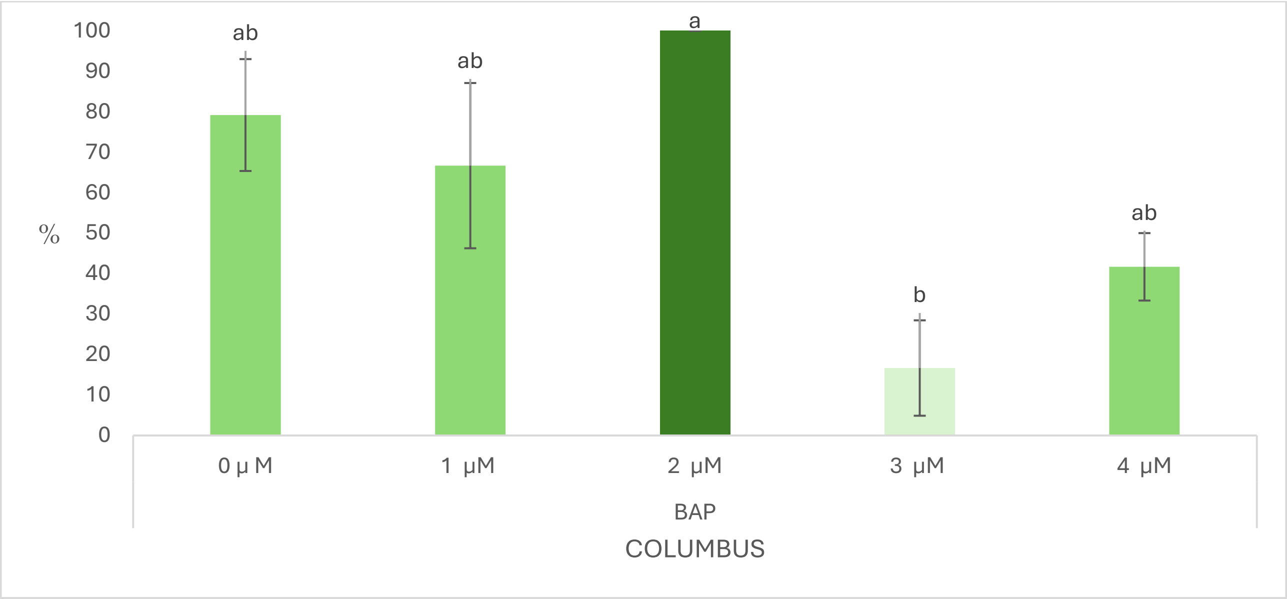


**Figure S2**

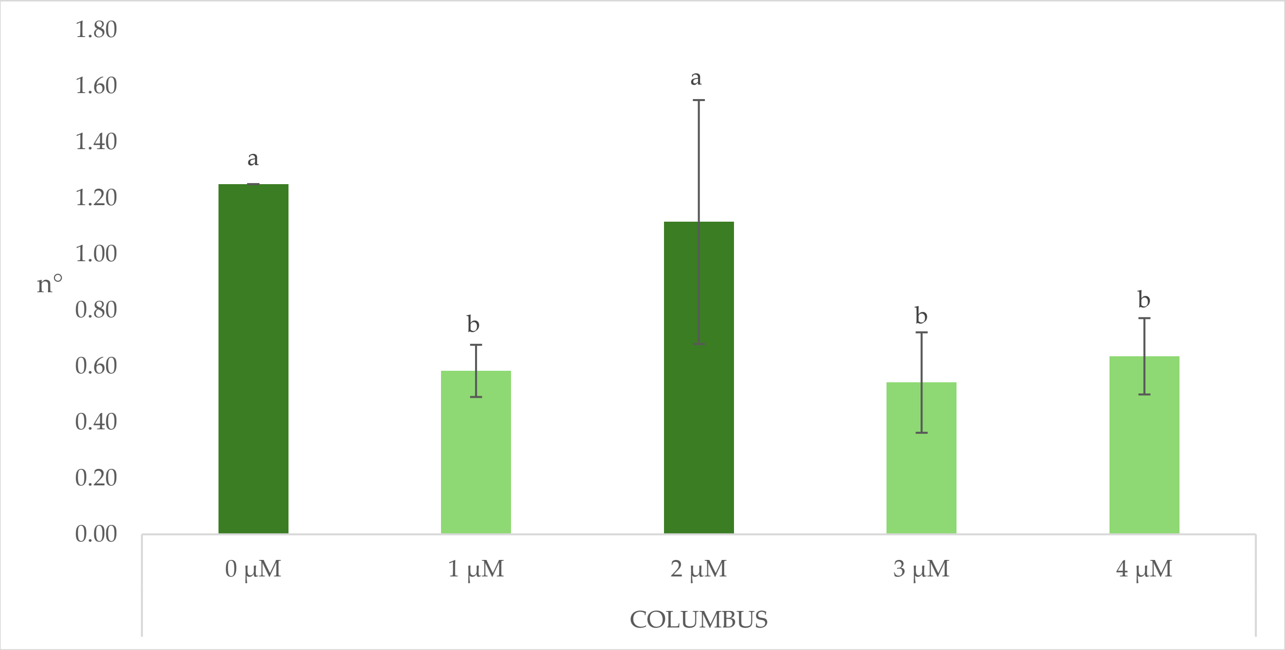


**Figure S3**

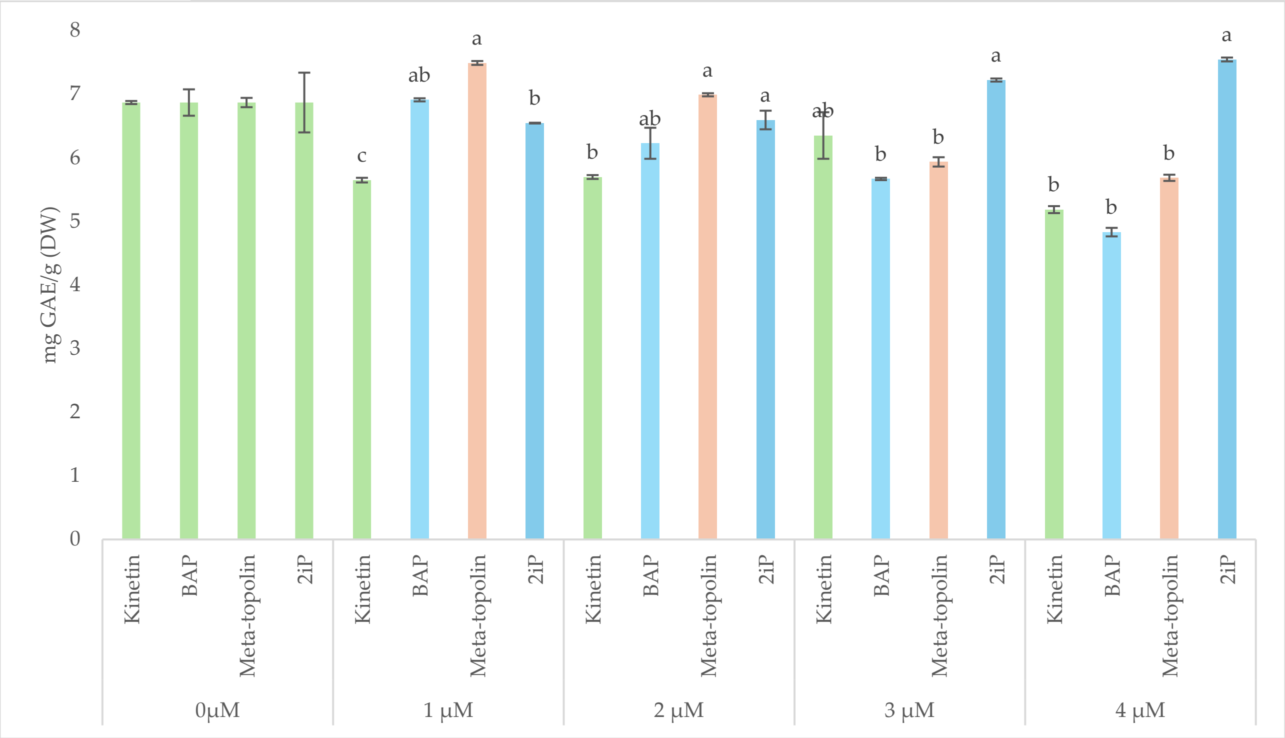


**Figure S4**

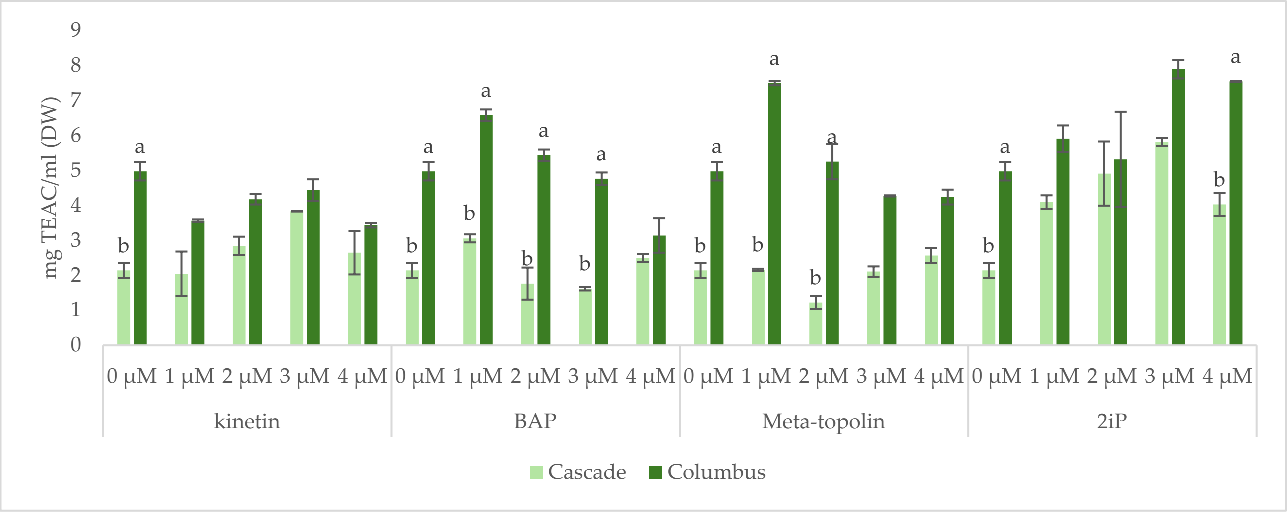


**Figure S5**

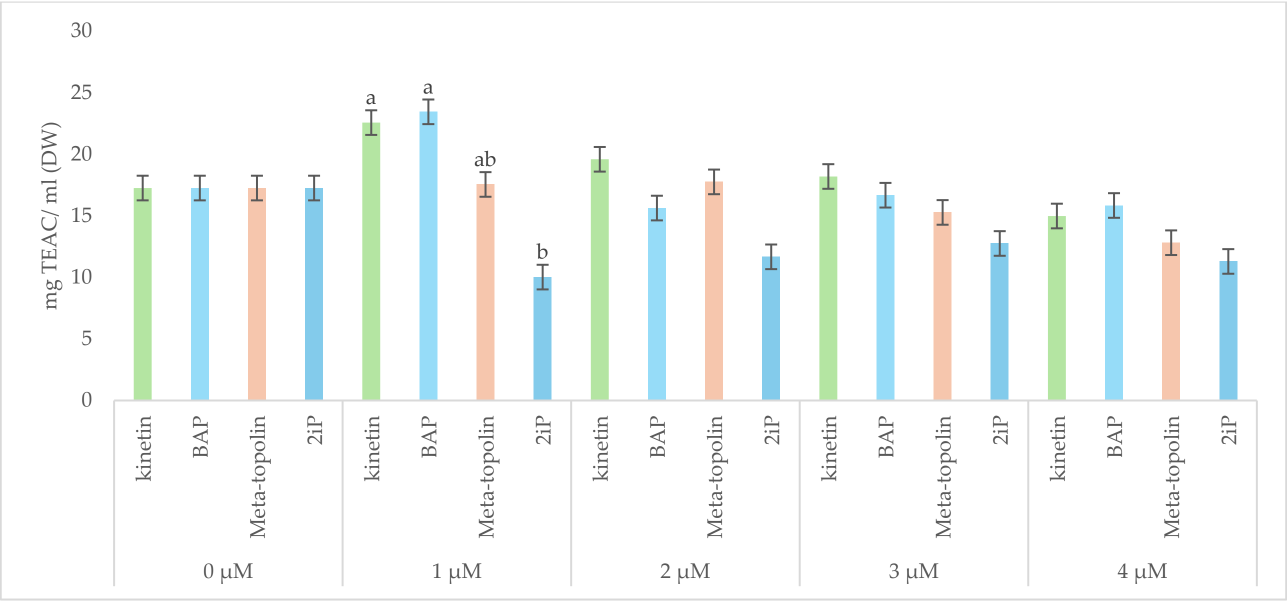


**Figure S6**

**Figure legends**

**Figure S1:** Effect of 'type of cytokinin' on the viability of cv. Cascade explants after four weeks of culture. Letters indicate statistically significant values according to the Tukey test post-two-way-ANOVA ('Genotype' × 'Growth Regulator') at $p \leq 0.05$ (Table 1). Abbreviations: BAP: 6-Benzylaminopurine; 2iP: 6-($\gamma$,$\gamma$-Dimethylallylamino)purine.

**Figure S2:** Effect of BAP concentration on the percentage of sprouted explants of cv. Columbus explants after four weeks of culture. Letters indicate statistically significant values according to the Tukey test post-three-way-ANOVA ('Genotype' × 'Growth Regulator'× 'Growth Regulator Concentration') at $p \leq 0.05$ (Table 1). Abbreviations: BAP: 6-Benzylaminopurine.

**Figure S3:** Effect of the concentration of the growth regulators tested on the number of sprouts produced by each explant of cv. Columbus after four weeks of culture. Letters indicate statistically significant values according to the Tukey test post-two-way-ANOVA ('Genotype' × 'Growth Regulator Concentration') at $p \leq 0.05$ (Table 1).

**Figure S4:** Effect of the type and concentration of the growth regulators tested on the total (poly)phenolic content of cv. Columbus plantlets after four weeks of culture. Letters indicate statistically significant values according to the Tukey test post-three-way-ANOVA ('genotype' × 'Growth Regulator'× 'Growth Regulator Concentration') at $p \leq 0.05$ (Table 2). Abbreviations: BAP: 6-Benzylaminopurine, 2iP: 6-($\gamma$,$\gamma$-Dimethylallylamino)purine.

**Figure S5:** Effect of the type and concentration of the growth regulators tested on antioxidant capacity (measured with DPPH assay) of cvs. Cascade and Columbus plantlets after four weeks of culture. Between the two genotypes tested, letters indicate statistically significant values, according to the Tukey test post-three-way-ANOVA ('Genotype' × 'Growth Regulator'× 'Growth Regulator Concentration') at $p \leq 0.05$ (Table 2). Abbreviations: BAP: 6-Benzylaminopurine, 2iP: 6-($\gamma$,$\gamma$-Dimethylallylamino)purine, DW: dry weight.

**Figure S6:** Effect of the type and concentration of the growth regulators tested on antioxidant capacity of cvs. Cascade and Columbus plantlets after four weeks of culture. Between the two genotypes tested, letters indicate statistically significant values, according to the Tukey test post-two-way-ANOVA ('Growth Regulator'× 'Growth Regulator Concentration') at $p \leq 0.05$ (Table 2). Abbreviations: BAP: 6-Benzylaminopurine, 2iP: 6-($\gamma$,$\gamma$-Dimethylallylamino)purine, DW: dry weight.
